# Computer-Assisted Avatar-Based Treatment for Dysfunctional Beliefs in Depressive Inpatients: A Pilot Study

**DOI:** 10.3389/fpsyt.2021.608997

**Published:** 2021-07-15

**Authors:** Martin Kocur, Martin Dechant, Christian Wolff, Caroline Nothdurfter, Thomas C. Wetter, Rainer Rupprecht, Youssef Shiban

**Affiliations:** ^1^Chair for Media Informatics, University of Regensburg, Regensburg, Germany; ^2^Department of Computer Science, University of Saskatchewan, Saskatoon, SK, Canada; ^3^Department of Psychiatry and Psychotherapy, University of Regensburg, Regensburg, Germany; ^4^Department for Clinical Psychology, Private University of Applied Sciences Göttingen, Göttingen, Germany

**Keywords:** modification of dysfunctional beliefs, cognitive therapy, avatar therapy, virtual avatar, depression

## Abstract

Dysfunctional cognitions are a crucial part of depression. Cognitive therapy aims to modify dysfunctional beliefs. Typically, dysfunctional beliefs are questioned, and patients are trained to think of alternative functional beliefs. We developed a computer-assisted, avatar-based adjunct for cognitive therapy that aims to reduce dysfunctional beliefs and symptom severity. Besides, it aims to promote alternative functional beliefs. In a randomized controlled trial with 34 patients diagnosed with major depression currently undergoing inpatient treatment at the university psychiatric hospital in Regensburg, Germany, participants were randomly assigned to receive either treatment as usual (TAU) or computer-assisted avatar-based treatment for dysfunctional beliefs (CAT-DB) in addition to TAU. In CAT-DB participants are faced with a virtual avatar expressing their personal dysfunctional beliefs. Participants are asked to contradict these and express alternative functional beliefs. Assessments of conviction of dysfunctional beliefs, functional beliefs and symptom severity were done shortly before the intervention (pre-treatment), right after the intervention (post-treatment) and 14 days later (follow-up). The reduction in conviction of dysfunctional beliefs and symptom severity, and the increase in conviction of alternative functional beliefs at post-treatment and follow-up were significantly greater for the group receiving CAT-DB. Our study provides an indication in favor of the effectiveness of CAT-DB for depressive patients. It is a simple tool that could support classical cognitive therapy. Further studies at different centres, with larger sample sizes and varying therapeutic contexts are required to prove the effectiveness of our intervention.

## Introduction

Major Depressive Disorder (MDD) is a burdensome mental disorder characterized by depressed mood, as well as a loss of interest and pleasure (1). It is associated with a variety of emotional, cognitive, physiological/vegetative and behavioral symptoms leading to considerable impairments in daily functioning (1, 2). There are various theories on the development and maintenance of this disorder from a broad range of perspective, such as inter alia, biological [for an overview, please see (3)] and psychological [e.g., (4–6)] models, as well as a diathesis-stress perspective [e.g., (7)]. One of the most prominent psychological models is Beck's cognitive theory of depression (4). According to Beck's theory, dysfunctional cognitive schemas are thought to cause a negatively biased view of reality as well as other depressive symptoms (such as anhedonia and motivational problems). Those schemas/patterns play a major role in the development and maintenance of depression (8–10). Schemas are stable cognitive structures representing an individual's experiences, which can remain dormant for many years (11). In depressive individuals, these schemas typically include themes of hopelessness, self-criticism and low self-esteem (2). They affect the way in which humans interpret, categorize and evaluate experiences (11). Furthermore, they underlie an individual's attitudes and core beliefs, which in the case of depression, are characterized by the so-called cognitive triad: Negative views of the self, the world and the future (2, 11, 12). For example, depressive individuals can hold a dysfunctional belief such as “I am unlovable.” The way in which they interpret experiences is, therefore, negatively distorted and biases how they process information encountered in daily lives. Information appearing to be consistent with such core beliefs receive greater attention whereas belief-inconsistent information is ignored. Furthermore, these dysfunctional beliefs can generate intermediate beliefs (“If I am fit and thin, I will be lovable”), which in turn elicit negative automatic thoughts (“I am obese and ugly”) as one of the primary cause of depressive symptoms (2, 8, 10, 11). These automatic thoughts are mirrored by absolute, distorted, illogical and inappropriate conclusions, in turn resulting in a reinforcement of the dysfunctional beliefs (2).

Beck's cognitive model provides the theoretical framework for cognitive therapy (13), one of the most prominent psychological treatment approaches for depression (10). Based on the theoretical background of Beck's theory, cognitive therapy aims to modify dysfunctional beliefs (thus debilitating depressogenic dysfunctional core beliefs) and enhancing more functional ones (10, 11). During cognitive therapy, patients learn to detect dysfunctional beliefs and to assess whether these cognitions are correct and useful (11). In addition, they are trained to think of more functional beliefs and, through homework in the form of experimentation, to examine the accuracy and usefulness of these new cognitions (11).

Recently, cognitive therapy and web-based self-help programs based on Beck's cognitive therapy were successfully used in the treatment of depression (14, 15). As previous research already suggests, internet-based interventions have some advantages over traditional approaches, like the increased accessibility of treatment, the ability to scale and the ease of access (16–18). Due to the rapid advance in technology, conversational interfaces have emerged and become widespread in recent years. Conversational agents (CAs), such as the popular Amazon's Alexa or Apple's Siri, allow the users to naturally interact with these applications in a way as if they would talk with a real human. Medical and psychological researchers have recognized the potential of this natural way of interaction with CAs to supplement or replace mental health services (19). Fitzpatrick et al. (20), for example, used a fully automated text-based CA known as “Woebot” running over a mobile app or Facebook messenger to deliver self-help based on cognitive behavioral therapy to students suffering from anxiety and depression. The authors found that the application of their mental health chatbot could reduce anxiety and the symptoms of depression. Similarly, Suganuma et al. (21) showed a high acceptability of an automated CA with a graphical representation enabling the users to communicate over a text-based dialogue. From a technical perspective, these CAs are artificial intelligent chatbots using simple text dialogues enabling the user to interact with such applications. More advanced embodied CAs, e.g., anthropomorphic 3D virtual characters capable of communicating verbally and non-verbally through facial expressions and gestures, have hardly been explored even if they could enhance the overall experience through a more authentic, believable and immersive communication (22). DeVault et al. (23), for example, created an autonomous virtual human interviewer capable of understanding simple natural language and non-verbal behavior. The authors showed that users experienced rapport and feelings comparable to a face-to-face interview. This is in line with Meeker et al. (24), who found that participants had a satisfying experience during their gamified intervention using a virtual human known as “SimCoach.” There is also evidence that such embodied CAs can reduce stress and fear connected with the perception of being judged (25). Likewise, Philip et al. (26) concluded that embodied CAs could even create a sense of trust, which increases the participants' willingness to interact with these virtual humans. Research from human-computer interaction even found that the appearance of virtual characters can affect the users' cognitive as well as physical performance and, therefore, may be leveraged to create effective health interventions (27–30). Despite all these promising findings, realistic 3D virtual agents are still in its infancy and, therefore, it is unknown whether a higher fidelity of a CA contributes to a higher therapeutic efficacy.

In a review about CAs in clinical psychology, Provoost et al. (31) concluded that the findings from studies conducted to date seem promising regarding its application and acceptability. Particularly, the increased access to mobile devices and the internet has made CAs attractive in recent years, as they can deliver autonomous mental health support. However, this autonomy during unguided intervention entails risks. Beside their decreased adherence (32), these applications are still based on algorithms not being able to understand the users' needs. As current autonomous CAs such as “Woebot” uses simple decision trees with only a limited number of responses, it is impossible that they cover, for example, the unlimited variety of dysfunctional beliefs underlying depressions. Hence, autonomous CAs are hardly applicable for tailored and personalized therapies with the aim to modify highly-individual dysfunctional beliefs. This in combination with the lack of comprehensive evidence for their efficacy in clinical populations makes on-site human support necessary (33).

Besides the usage of web-based technology, also computer generated virtual environments and immersive technologies, such as virtual reality (VR) are used as part of a treatment: The *Clinical VR—*the use of interactive 3D virtual environments for clinical purposes (34)—has been successfully applied to reduce symptoms in different phobias (35, 36) and depression (37). According to Rizzo and Koenig (34), Clinical VR applications are “indispensable tools in the toolbox of psychological researchers” and have several advantages over common *in-vivo* or in-sensu therapies: First, *in-vivo* exposition are always limited to the real environment and often involve larger logistical and financal efforts to recreate a specific situation for the patient and the therapist (38). In-virtuo exposure cuts these costs, since the patient stays in a save virtual environment, which can be adjusted to the requirements of the therapy. Rizzo et al. (39), for example, recreated a virtual war scenario in the *Virtual Iraq* environment for veterans suffering from PTSD to expose them to their traumatic experiences thus helping the patient to modify and emotionally process pathological and dysfunctional structures. Besides the financial and logistical aspects, in-virtuo gives the therapist the complete control over the perceived reality for the patient. This allows to optimize and adjust the exposure for the patients' personal needs. Furthermore it enables the therapist to stop the exposure immediately. This level of control is very difficult to achieve in a real world environment. Therefore in-virtuo lowers the risks for both, the patient and the therapist (40).

Another aspect is the user acceptance and the success of in-virtuo vs. *in-vivo* exposure: according to Botella et al. (41), patients showed a higher treatment acceptance and a greater willingness to address their fears while being exposed to a fearful situation in a safe virtual environment. Additionally, Jaycox et al. (42) showed that restricted emotional engagement in imagination is a predictor for negative treatment outcome. Thus, patients with problems imagining demanding situations or anxiety triggering events could face an advantage by undergoing in-virtuo instead of in-sensu trainings since in-virtuo does not rely on the patients' imagination. However, the efficacy of such techniques to reduce dysfunctional beliefs and to promote alternative beliefs for people with major depression is not clearly evaluated. Therefore, to leverage the benefits of the previously introduced technologies to increase the efficacy of treatments, we propose an avatar-based adjunct, which should support classical cognitive therapy approaches.

This program is hereafter referred to as CAT-DB (computer-assisted avatar-based treatment for dysfunctional beliefs). CAT-DB was inspired by the work of Craig et al. (43), who investigated the efficacy of a so-called avatar therapy [for a detailed description of avatar therapy, see (44)] for reducing auditory verbal hallucinations in individuals suffering from psychosis. During avatar therapy, participants have a conversation with a human like avatar representing their supposed persecutor (43). They must assert themselves in the discussion, with the avatar confronting them with the contents of their hallucinations in the beginning of the intervention but gradually losing ground during the treatment (43). Craig et al. (43) showed that this avatar therapy was successful in diminishing the severity of patients' auditory verbal hallucinations.

Similar to the previously introduced avatar therapy by Craig et al. (43) representing persecutory auditory hallucinations, CAT-DB involves presenting participants a virtual avatar embodying the individual dysfunctional beliefs. As beliefs are an abstract construct, which have no representation in the physical world and, therefore, neither physical nor spatial constraints, it is complex to understand what they actually are. To simplify the complex and abstract nature of beliefs and make them “tangible,” we therefore use our virtual avatar to represent them as a human entity. Consequently, this should enable the patients to have a dialogue with their dysfunctional beliefs. Unlike avatar therapy for psychotic patients, CAT-DB is used as a treatment method for depressive individuals. The virtual avatar therefore confronts the individual with their dysfunctional beliefs, such as “You have to be perfect.” The participants use individual alternative beliefs to contradict the virtual avatar, like “No, that's not true. Everybody makes mistakes.” Similar to the rationale of attention training techniques (45), CAT-DB should therefore redirect the participants' biased attention to negative beliefs and thoughts toward alternative functional ones. Through the repeated generation of alternative functional beliefs, we aim at initiating a cognitive restructuring process by shifting the activation of functional beliefs from a cognitively demanding controlled process, i.e., the participants have to actively stop unwanted dysfunctional thoughts and activate functional ones, to an automatic process, i.e., functional beliefs are automatically elicited in certain situations, that can counteract the dysfunctional beliefs.

The objective of the present pilot study was to investigate whether a three-session CAT-DB would be capable of reducing dysfunctional cognitions and promoting functional alternative beliefs in depressive patients. Additionally, this study aimed to investigate whether the three-session CAT-DB can reduce symptom severity. Therefore, we examined a sample of depressive inpatients, randomly assigning them either to a group receiving three sessions of CAT-DB in addition to inpatient treatment as usual (TAU)—hereafter referred to as the CAT-DB+TAU group—or to a control group undergoing TAU but not receiving CAT-DB—hereafter referred to as the TAU group. Throughout the study, the participants repeatedly rated how convinced they were that their dysfunctional and alternative beliefs were true.

We hypothesized that, in comparison to the TAU group, the CAT-DB+TAU group would result in decreased conviction ratings for the individual dysfunctional beliefs and increased conviction ratings for the individual alternative functional beliefs from pre- to post-treatment, as well as from pre-treatment to follow-up (2 weeks later). Finally, we expected that, compared to the TAU group, the CAT-DB+TAU group would show a greater decrease in symptom severity from pre- to post-treatment and from pre-treatment to follow-up.

## Materials and Methods

### Ethics

Written informed consent was obtained from the individual(s) for the publication of any potentially identifiable images or data included in this article. The study was approved by the Ethics Committee of the University of Regensburg, Germany (number: 17-784-101). Furthermore, the trial was prospectively registered with ClinicalTrials.gov, number NCT03389464.

### Participants

**Figure 1:**
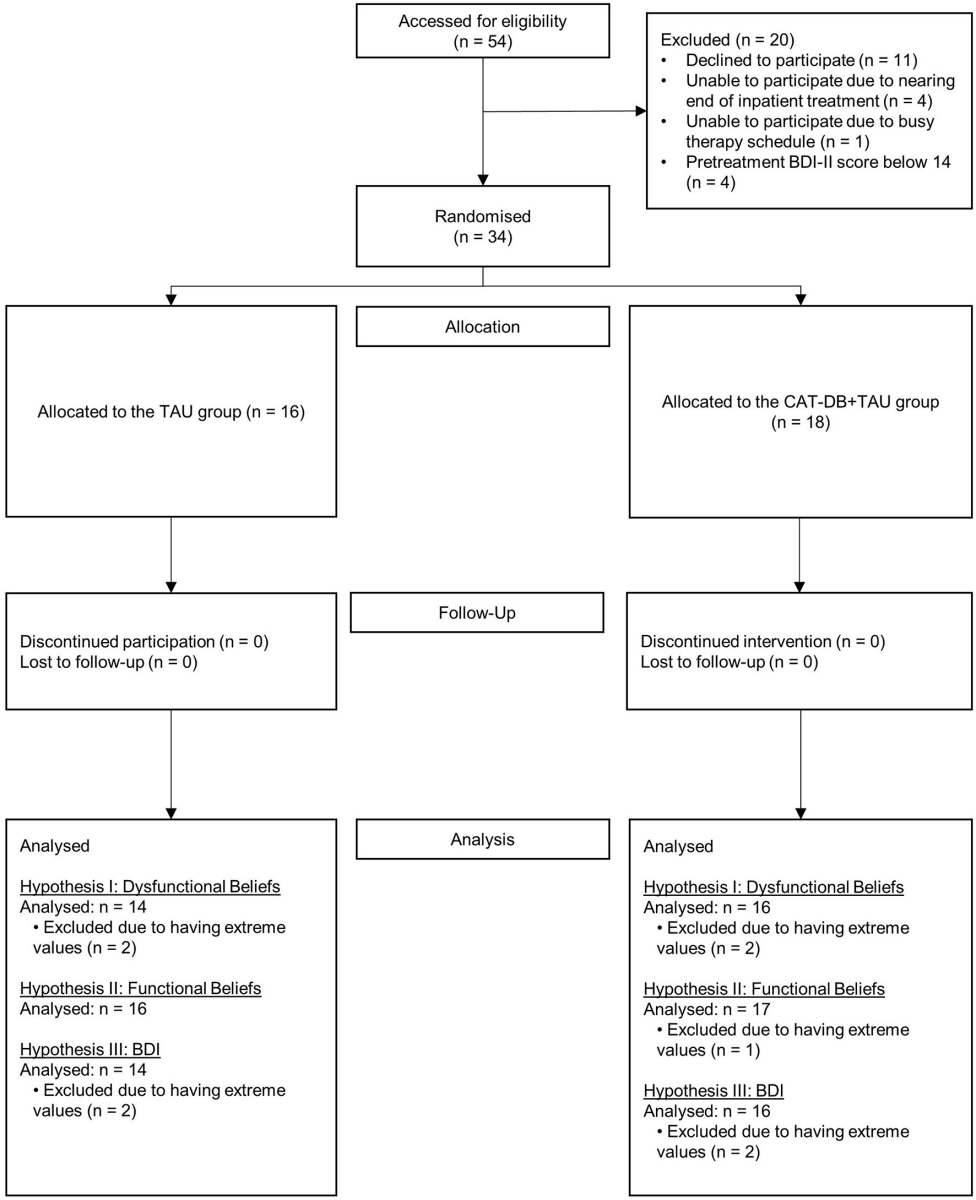
Participant flow chart.

We recruited 54 individuals (30 women, 24 men; *M*_age_ = 39, *SD*_age_ = 14.41, age range: 20–63) diagnosed with major depression and currently undergoing inpatient treatment at the university psychiatric hospital in Regensburg, Germany. Exclusion criteria included substantial and imminent suicide risk, current or history of psychotic symptoms, current manic episode, hearing problems, poor eyesight, pregnancy and age under 18 or above 65 years. 16 participants had to be excluded or dropped out of the study due to organizational reasons. Additionally, to ensure that participants were at least suffering from “mild” depression, we excluded four participants with a pre-treatment BDI-II score below 14 (for further information on the sum score of the BDI-II, please see the materials section). Thus, this study is based on the data from 34 inpatients (21 women, 13 men; *M*_age_ = 37, *SD*_age_ = 12.68, age range: 20–59). We had no discontinued interventions and drop-outs from allocation to follow-up. At follow-up, eight participants (four from each group) had already been discharged from the psychiatric hospital. All of them filled in the follow-up questionnaires and documents at home and returned them to the study. Figure 1 shows the participant flow throughout the study.

For the 34 individuals included in the present study, the average pre-treatment BDI-II score was 30.38 (*SD* = 7.96, range: 14–46). Participating in the study, these patients had already spent an average of 4.24 (*SD* = 2.73, range: 1–11) weeks at the psychiatric hospital in Regensburg. We assigned a number to each participant and used a computer algorithm, which randomly allocated the participants to the CAT-DB+TAU group (12 women, 6 men) or the TAU group (9 women, 7 men). The two groups did not differ significantly with respect to age, age of onset, number of depressive episodes, duration of index episode, time since admission, days until follow-up or the scores obtained in any of the pre-treatment measurements (see Table A.1 in Appendix A).

### Study Design and Outcomes

The study was subdivided into five sessions: one pre-treatment session, three CAT-DB sessions for those in the respective group and one post-treatment session taking place on consecutive days (Monday–Friday) at the university psychiatric hospital in Regensburg, Germany. Furthermore, there was a follow-up session 2 weeks after the post-treatment session. TAU was provided to both groups and was according to the relevant local health systems and practices including psychopharmacological therapy, e.g., SSRI or atypical neuroleptic, scheduled individual and group therapy as well as additional appointments if needed.

At the pre-treatment session, participants completed a demographic questionnaire as well as the German Version of the BDI-II [BDI-II; (46); German version: (47)]. The BDI-II was used to assess symptom severity. In accordance with the experimenter explained the basic concept of dysfunctional beliefs as cognitive distortions, which are not mainly specific to certain situations (e.g., “I have to be perfect at the next exam.”) but are based on general beliefs (e.g., “I have to be perfect.”). Afterwards, the participants were asked to think of and write down three individual dysfunctional beliefs. Then, the experimenter explained the concept of alternative beliefs to the participants and asked them to think of and write down an alternative belief (e.g., “Everybody makes mistakes.”) for each of their three dysfunctional beliefs. The experimenter provided support in formulating the beliefs if necessary by explaining that they should think about reasons why they feel sad and depressed. The participants were asked to give a conviction rating for each of their dysfunctional and alternative beliefs, i.e., to indicate (on a scale from 0 = “not convinced at all” to 100 = “absolutely convinced”) how convinced they were that the respective dysfunctional/alternative belief was true.

The experimental setup consisted of two laptops, one for the experimenter and one for the participant (Intel® Core™ i5-7200U, 8 GB RAM, resolution: 1920 × 1080).

**Figure 2:**
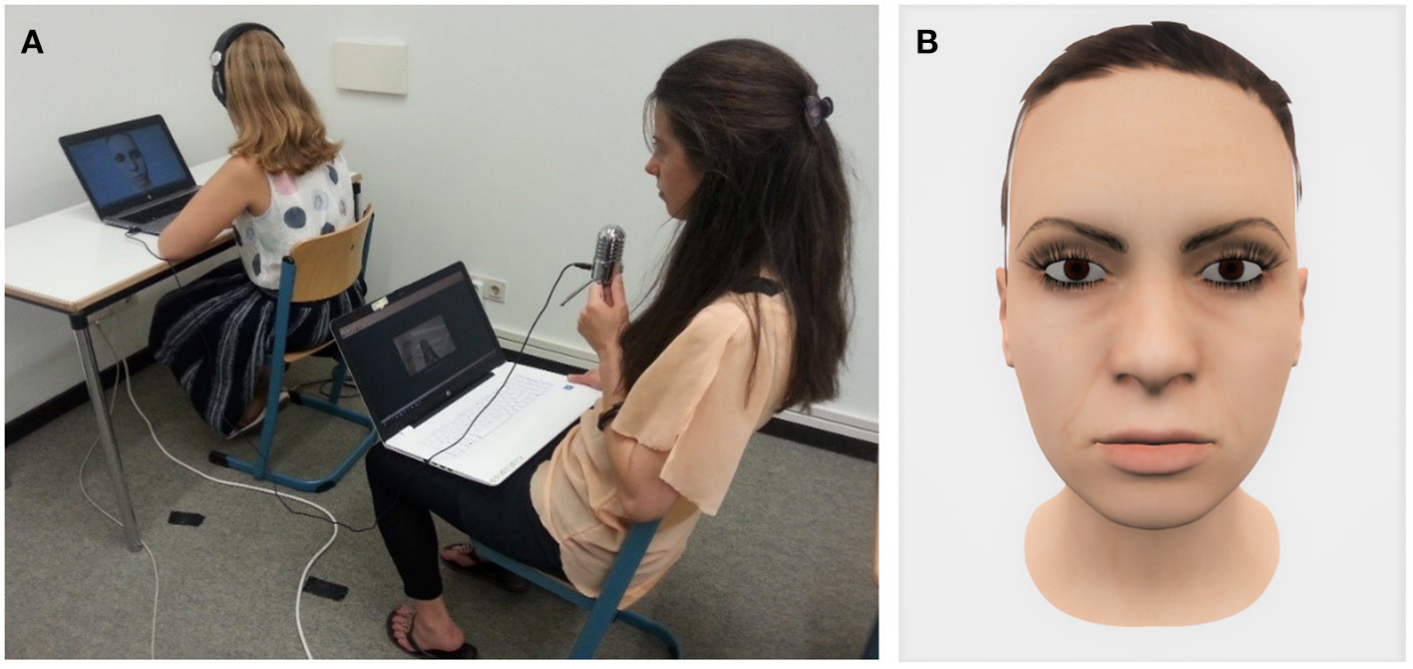
Experimental setup **(A)** and screenshot of the virtual avatar **(B)**. The participant sat in front of a computer screen displaying the virtual avatar. Via headphones, the participant could hear the experimenter speaking into a microphone. Throughout CAT-DB, the experimenter's facial expressions and lip movements were captured and translated onto the virtual avatar's face in real time.

During the CAT-DB sessions the respective participants wore earphones (Samson Meteor Mic, Samson AG, Germany) and sat in front of a connected desktop screen, which displayed a female virtual avatar. The left panel of Figure 2 shows a recreation of the experimental setup; the right panel of Figure 2 shows the used female virtual avatar.

During CAT-DB, the female virtual avatar's face was presented to participants on a laptop screen. By using a second laptop and the software Faceware Live (FacewareTechnologies Inc.), the experimenter's facial expressions were captured and translated onto the virtual avatar's face in real time. CAT-DB consisted of 30 confrontations (10 confrontations per dysfunctional belief) with a short break after the first 15 confrontations. The order in which the dysfunctional beliefs were presented was the same throughout the entire session (dysfunctional belief 1—dysfunctional belief 2—dysfunctional belief 3—dysfunctional belief 1—…). As soon as the participants had contradicted the virtual avatar, the next confrontation followed immediately. At post-treatment and follow-up, the participants filled in the BDI-II and gave conviction ratings for each of their dysfunctional and each of their alternative beliefs again.

The basic shape of a female virtual avatar was created with the open source tool MakeHuman (48) and exported to the open source 3D creation suite Blender (49), which was used to model the avatar and to create facial expressions. The virtual world was implemented using the Unreal Engine (50).

### Statistical Analysis

Data analyses were performed with IBM Corp (51). Based on the conviction ratings given by the participants for each of their three dysfunctional beliefs, a mean score of the conviction ratings for the dysfunctional beliefs for each session was calculated. In the same manner a mean score of the conviction rating for the alternative functional beliefs was calculated. Kolmogorov-Smirnov tests for normality were used to test the assumption of normal distribution for parametric data.

The BDI-II scores, the mean scores of the conviction ratings for the dysfunctional beliefs and the mean scores of the conviction ratings for the alternative beliefs were submitted to a 2 × 3 repeated-measures ANOVA with the between-subjects factor group (CAT-DB+TAU group, TAU group) and the within-subjects factor session (pre-treatment, post-treatment, follow-up) each. If required, we adjusted the degrees of freedom of the F-statistics by multiplying them with the Greenhouse–Geisser estimate to correct for deviations from spherical data.

Each ANOVA is followed by the same set of *post-hoc t*-tests. All *post-hoc t*-tests were Bonferroni corrected. First, for both groups separately we tested whether the mean of an outcome is different at pre-treatment from post-treatment as well as at pre-treatment from follow-up. These tests helped to discriminate how each group contributes to potential session effects. Second, the differences of an outcome from pre-treatment to post-treatment as well as from pre-treatment to follow-up were calculated. Then *t*-tests were used to compare whether the means of these differences differ significantly between the two groups. These *t*-tests were useful to analyse potential Group × Session interaction effects and further explore whether both groups evolve differently over the course of time. In our analyses we removed extreme values, as described below, that can be regarded as outliers. In Appendix B we report all analyses without removing any outliers.

## Results

### Conviction Ratings for the Dysfunctional Beliefs

Descriptive analysis revealed that four participants had extreme dysfunctional belief scores (their score was outside the interval spanned by subtracting and adding 1.5 times the interquartile range to the first and third quartile). These participants were excluded from the statistical analysis.

**Figure 3:**
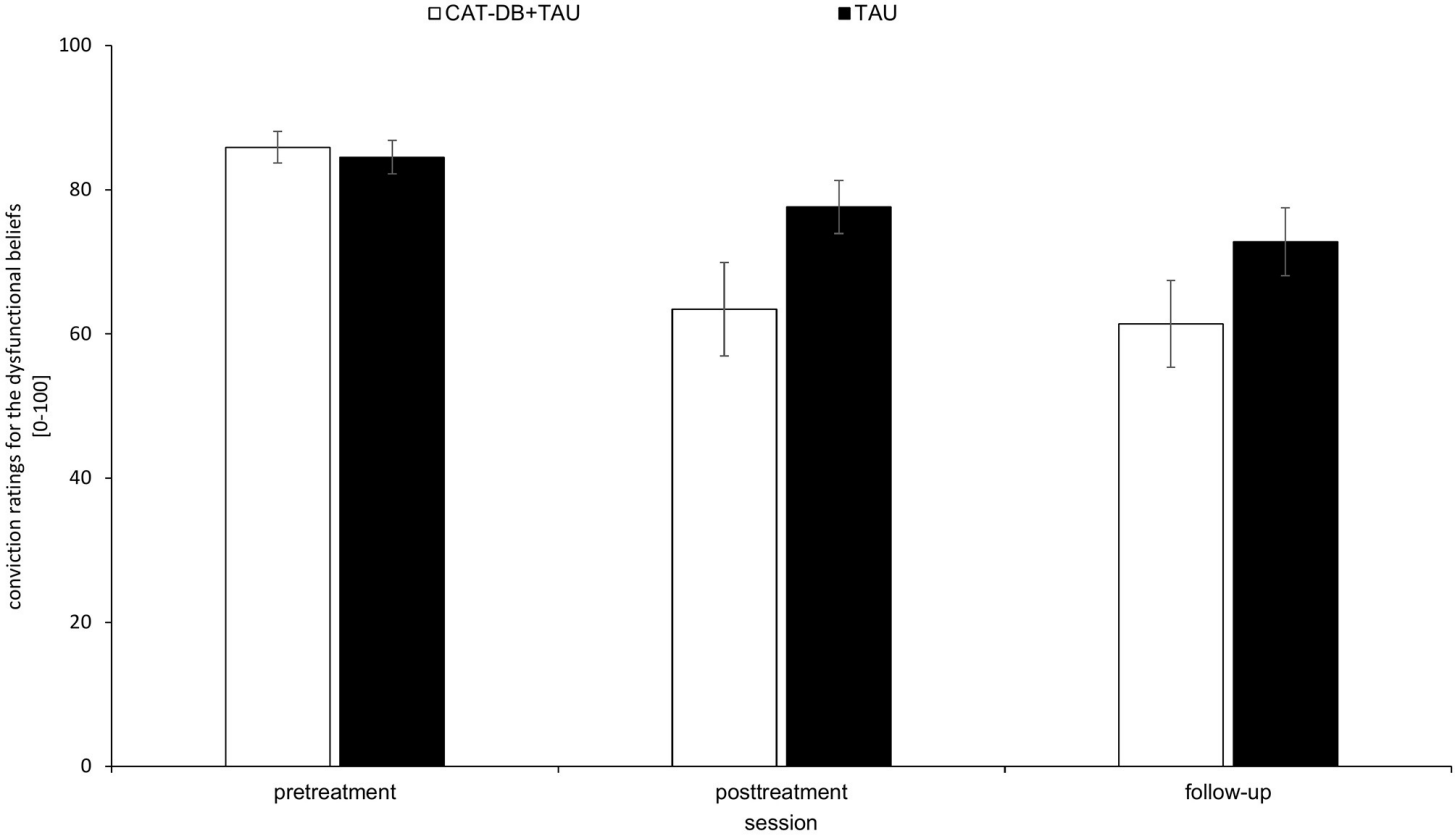
Mean score of the conviction ratings for the dysfunctional beliefs in the CAT-DB+TAU group (*n* = 16) and the TAU group (*n* = 14) at pre-treatment, at post-treatment and at follow-up. Error bars represent standard errors.

As shown in Figure 3, there was a decrease in the mean score of the conviction ratings for the dysfunctional beliefs between pre-treatment and post-treatment, as well as from post-treatment to follow-up in both groups, with a more pronounced decrease in the CAT-DB+TAU group than in the TAU group.

A 2 × 3 repeated-measures ANOVA with the between-subjects factor group (CAT-DB+TAU group, TAU group) and the within-subjects factor session (pre-treatment, post-treatment, follow-up) revealed a significant effect for Session, *F*_(1.696, 47.495)_ = 22.873, *p* < 0.001, η^2^ = 0.45 and for the Group × Session interaction, *F*_(1.696, 47.495)_ = 4.249, *p* = 0.025, η^2^ = 0.132. There was no significant effect for Group, *F*_(1, 28)_ = 1.996, *p* = 0.169, η^2^ = 0.067. Paired *post-hoc t*-tests for both groups revealed that the mean scores of the conviction ratings of the following sessions differed significantly from each other: For the TAU group the mean score of the conviction ratings given at pre-treatment (*M* = 84.55, *SD* = 8.75) was significantly higher than the mean score of the conviction ratings given at follow-up (*M* = 72.79, *SD* = 17.49), *p* = 0.036. For the CAT-DB+TAU group the mean score of the conviction ratings given at pre-treatment (*M* = 85.92 *SD* = 8.74) was significantly higher than the mean score of the conviction ratings given at post-treatment (*M* = 63.41, *SD* = 25.88), *p* < 0.001, and the mean score of the conviction ratings given at follow-up (*M* = 61.42, *SD* = 24.01), *p* < 0.001.

We performed a *t*-test to analyse the means of differences from pre-treatment to post-treatment. We found significant differences between the TAU group (*M* = 6.90*, SD* = 10.33) and the CAT-DB+TAU group (*M* = 22.5*, SD* = 22.25), *t*(28) = −2.402, *p* = 0.023, *d* = 0. Additionally, the means of the difference from pre-treatment to follow-up differed significantly between the TAU group (*M* = 11.76*, SD* = 12.36) and the CAT-DB+TAU group (*M* = 24.5*, SD* = 19.03), *t*(28) = −2.123, *p* = 0.043, *d* = 0.78.

To explore and get first insights into potential adverse effects of CAT-DB, we descriptively analyzed the frequencies and percentages of patients with adverse treatment responses in terms of increased conviction ratings for dysfunctional beliefs (see Table B.4 in Appendix B). We found for the TAU group that two participants (12.50%) had increased conviction ratings for the dysfunctional beliefs from pre-treatment to post-treatment and from pre-treatment to follow-up. For CAT-DB+TAU group, we found that three participants (16.67%) had increased conviction ratings for the dysfunctional beliefs from pre-treatment to post-treatment and two participants (11.11%) from pre-treatment to follow-up.

### Conviction Ratings for the Functional Beliefs

Descriptive analysis revealed that one participant had extreme alternative belief scores (the score is outside the interval spanned by subtracting and adding 1.5 times the interquartile range to the first and third quartile). The participant was excluded from the statistical analysis.

**Figure 4:**
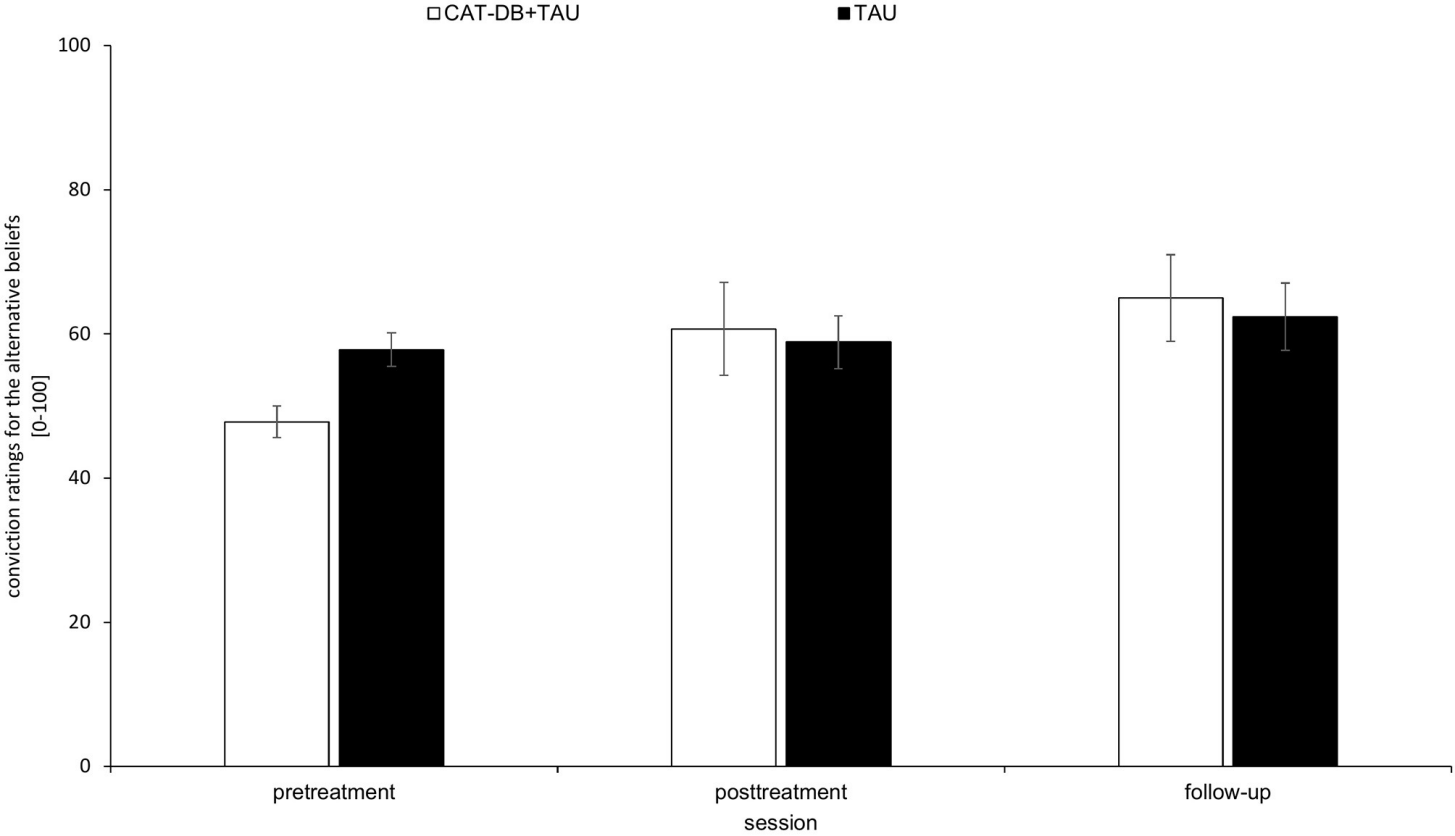
Mean score of the conviction ratings for the alternative beliefs in the CAT- DB+TAU group (*n* = 17) and the TAU group (*n* = 16) at pre-treatment, at post-treatment and at follow-up. Error bars represent standard errors.

As shown in Figure 4, in the CAT-DB+TAU group, there was an increase in the mean score of the conviction ratings for the alternative beliefs from pre-treatment to post-treatment and from post-treatment to follow-up. In the TAU group, there was a slight increase from pre-treatment/post-treatment to follow-up while the mean scores at pre-treatment and post-treatment were almost equal. The mean score of the conviction ratings was descriptively higher in the TAU group than in the CAT-DB+TAU group before the intervention. This difference is not significant (see Table A.1, Appendix A).

A 2 × 3 repeated-measures ANOVA with the between-subjects factor group (CAT-DB+TAU group, TAU group) and the within-subjects factor session (pre-treatment, post-treatment, follow-up) revealed a significant effect for Session, *F*_(1.672, 51.83)_ = 8.865, *p* = 0.001, η^2^ = 0.22 and for the Group × Session interaction, *F*_(1.672, 51.83)_ = 3.631, *p* = 0.041, η^2^ = 0.105. There was no significant effect for Group, *F*_(1, 31)_ = 0.055, *p* = 0.816, η^2^ = 0.002.

Paired *post-hoc t*-tests revealed that the mean scores of the conviction ratings of the following sessions differed significantly from each other for the CAT-DB+TAU group: The mean score of the conviction ratings given at pre-treatment (*M* = 47.82, *SD* = 29.52) was significantly lower than the mean score of the conviction ratings given at post-treatment (*M* = 60.68, *SD* = 28.30), *p* = 0.006, and the mean score of the conviction ratings given at follow-up (*M* = 65, *SD* = 23.93), *p* = 0.001. For the TAU group these comparisons did not reveal significant differences, *p* > 0.05.

We performed a *t*-test to investigate whether there were statistical differences between the means of differences from pre-treatment to post-treatment. We found a significant difference between the TAU group (*M* = −1.06*, SD* = 15.42) and the CAT-DB+TAU group (*M* = −12.86*, SD* = 16.17), *t*(31) = 2.143, *p* = 0.04, *d* = 0.75. Additionally, the means of the differences from pre-treatment to follow-up differed significantly between the TAU group (*M* = −4.58*, SD* = 15.82) and the CAT-DB+TAU group (*M* = −17.18*, SD* = 18.51), *t*(31) = 2.095, *p* = 0.044, *d* = 0.73.

### Symptom Severity: Beck Depression Inventory-II

Descriptive analysis revealed that four participants had extreme BDI-II sum scores (their score is outside the interval spanned by subtracting and adding 1.5 times the interquartile range to the first and third quartile). These participants were excluded from the statistical analysis.

**Figure 5:**
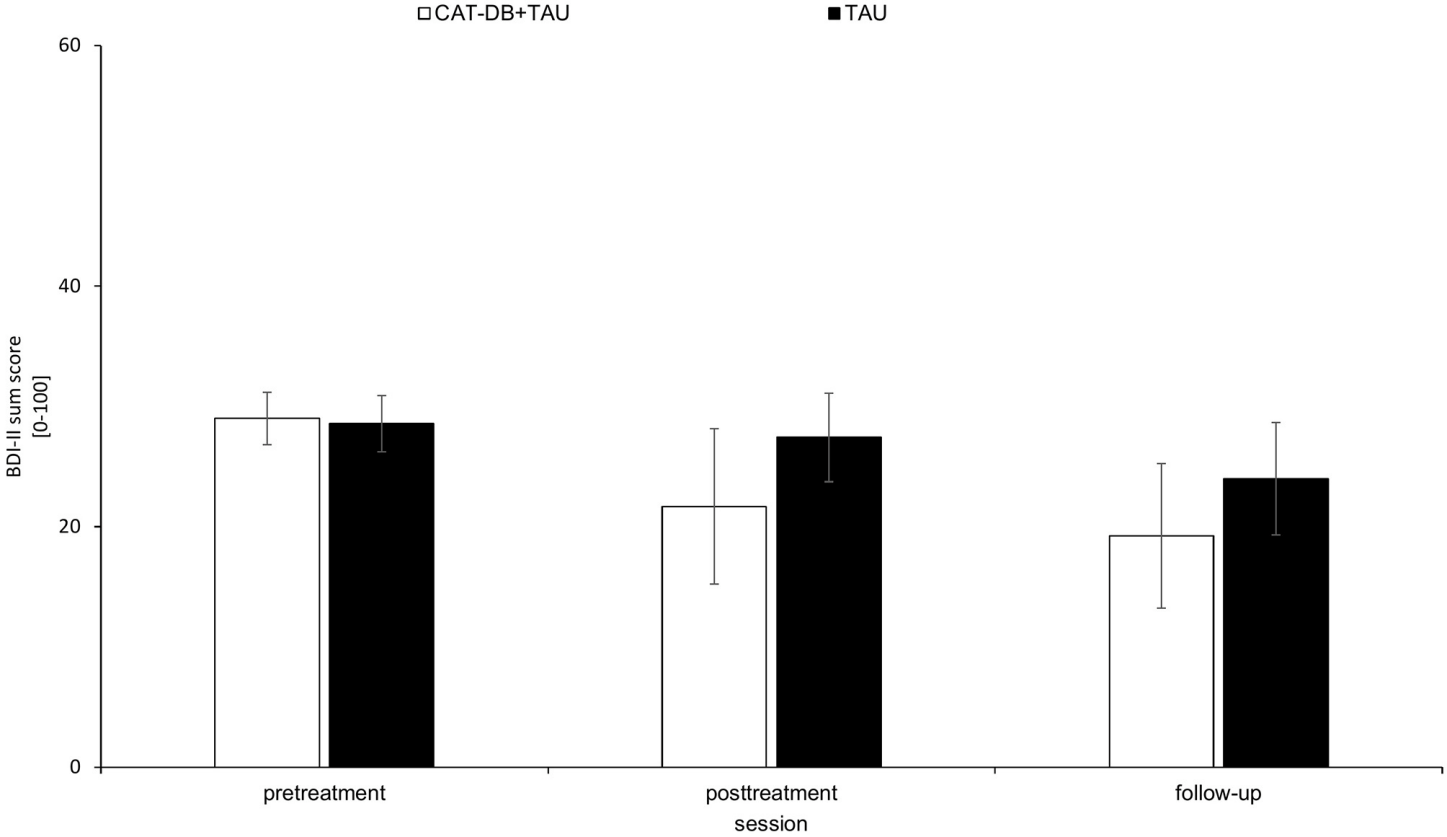
BDI-II sum score in the CAT-DB+TAU group (*n* = 16) and the TAU group (*n* = 14) at pre-treatment, at post-treatment and at follow-up. Error bars represent standard errors.

As shown in Figure 5, there was a decrease in the BDI-II sum score from pre-treatment to post-treatment and from post-treatment to follow-up in both groups, with larger effects in the CAT-DB+TAU group.

A 2 × 3 repeated-measures ANOVA with the between-subjects factor group (CAT-DB+TAU group, TAU group) and the within-subjects factor Session (pre-treatment, post-treatment, follow-up) revealed a significant effect for Session, *F*_(1.968, 55.115)_ = 20.137, *p* < 0.001, η^2^ = 0.418 and for the Group × Session interaction, *F*_(1.968, 55.115)_ = 4.265, *p* = 0.019, η^2^ = 0.132. There was no significant effect for Group, *F*_(1, 28)_ = 2.684, *p* = 0.113, η^2^ = 0.087.

Paired *post-hoc t*-tests for the TAU group revealed that mean BDI sum score given at pre-treatment (*M* = 28.57, *SD* = 6.69) was not significantly different from the mean BDI sum score given at post-treatment (*M* = 27.43, *SD* = 6.95), *p* > 0.050. Yet, for the TAU group the mean BDI sum score was significantly higher at pre-treatment than the mean BDI sum score given at follow-up (*M* = 24, *SD* = 3.98), *p* = 0.020. For the CAT-DB+TAU group the mean BDI sum score given at pre-treatment (*M* = 29 *SD* = 7.25) was significantly higher than the mean BDI sum score given at post-treatment (*M* = 21.67, *SD* = 8.32), *p* < 0.001, and the mean BDI sum score given at follow-up (*M* = 19.25, *SD* = 5.54), *p* < 0.001.

Additionally, we performed a *t*-test including the means of differences from pre-treatment to post-treatment. We found that the means of the differences from pre-treatment to post-treatment differed significantly between the TAU group (*M* = 1.14*, SD* = 4.99) and the CAT-DB+TAU group (*M* = 7.31*, SD* = 7.19), *t*(28) = −2.691, *p* = 0.012, *d* = 0.98. Additionally, a *t*-test revealed that the means of the differences from pre-treatment to follow-up differed significantly between the TAU group (*M* = 4.57*, SD* = 5.98) and the CAT-DB+TAU group (*M* = 9.75*, SD* = 5.67), *t*(28) = −2.431, *p* = 0.022, *d* = 0.89.

We report the frequencies and percentages of patients with adverse treatment responses in terms of increased BDI-II scores to explore potential negative effects of our intervention (see Table B.4 in Appendix B). We found for the TAU group that four participants (25.00%) had increased BDI-II scores from pre-treatment to post-treatment and from pre-treatment to follow-up. For CAT-DB+TAU group, we found that five participants (27.78%) had increased BDI-II scores from pre-treatment to post-treatment and three participants (16.67%) from pre-treatment to follow-up.

## Discussion

The objective of the present pilot study was to investigate the short-term efficacy of a three-session, computer-assisted, avatar-based intervention aimed at reducing dysfunctional beliefs and symptom severity as well as promoting functional alternative beliefs in depressive patients.

When removing outlier, the applied 2 × 3 repeated-measures ANOVAs revealed significant effects for the within factor Session for all three hypotheses. This is an expected result since all participants underwent inpatient treatment (including psychopharmacological therapy as well as individual and group therapy). Additionally, for all three hypotheses significant Group × Session interactions were found. Our results should be interpreted with caution and when outliers are kept, the applied ANOVAs reveal significant effects for Session for all three dependent variables, yet the Group × Session interaction is only significant for the Conviction Ratings for the functional beliefs (see Appendix B).

### Conviction Rating for the Dysfunctional Beliefs

The significant Group × Session interaction effect shows that both groups developed differently over the course of time. Both groups had gains from their treatment. However, the TAU condition only showed improvements from pre-treatment to follow-up whereas CAT-DB+TAU revealed a systematic decrease in conviction ratings from pre-treatment to post-treatment, and from pre-treatment to follow-up. Additionally, the larger effect sizes for the CAT-DB+TAU in the comparisons of pre-treatment with follow-up suggest that this group face greater gains. This assumption is supported by the significant results obtained by comparing the means of differences. The means of differences from pre-treatment to post-treatment as well as the means of differences from pre-treatment to post-treatment were significantly larger for the CAT-DB+TAU group. This means that the reduction in the conviction for dysfunctional beliefs was greater in the CAT-DB+TAU group than in the TAU group.

### Conviction Rating for the Functional Beliefs

Overall, inpatients increased their conviction rating for their functional beliefs over the course of time (significant main effect of session). Stratified for groups one can observe that this effect may be driven by the CAT-DB-group only: The comparisons of pre-treatment with post-treatment and pre-treatment with follow-up were only significant for CAT-DB+TAU group. This means that only inpatients of the CAT-DB+TAU group gained significant increases of the conviction ratings for their functional beliefs. Additionally, the mean of differences from pre-treatment to post-treatment as well as the mean of differences from pre-treatment to post-treatment were significantly larger for the CAT-DB+TAU group than for the TAU group. In other words, the average increase of conviction ratings for the functional beliefs was significantly larger for the CAT-DB+TAU group.

### Symptom Severity: Beck Depression Inventory-II

While the comparisons of means from pre-treatment to post-treatment as well as pre-treatment to follow-up were both significant for the CAT-DB+TAU group, for the TAU group only the comparison of means from pre-treatment to follow-up was significant. This means that the CAT-DB+TAU group faces significant decreases of their BDI scores earlier than the TAU group. Additionally, the significant interaction effect shows that both groups developed differently over the course of time. This is supported by the finding that the mean of differences from pre-treatment to post-treatment as well as the mean of differences from pre-treatment to post-treatment were significantly larger for the CAT-DB+TAU group. Inpatients of the CAT-DB+TAU group faced greater reductions of their BDI scores.

Taken together, the results obtained when removing outlier confirm all hypotheses stated above and therefore indicate that the intervention might be a useful tool for modifying dysfunctional beliefs in depressive inpatients. According to a *post-hoc* power analysis, our sample has sufficient power to reveal statistical significant differences with medium to large effects (Cohen's *d* = 0.73 and *d* = 0.98) between conditions at α = 0.05 with a power of 0.8. This is in line with findings from previous work investigating the efficacy of mental health interventions using virtual characters to treat depressions based on cognitive behavior therapy (31). Burton et al. (52), for example, also found that their embodied CA could serve as an adjunct for cognitive therapy. In contrast to our study focussing on dysfunctional beliefs as one of the main causes underlying depressive symptoms, the authors only used the BDI-II to assess the efficacy of their therapy. Likewise, recent meta-analyses demonstrated that embodied CAs delivering help programs are successful treatments (31, 53), particularly when being human-supported, e.g., due to an increased adherence (54). Although our intervention is guided and human-supported, it conceptually differs from conventional embodied CAs. As these agent-based interventions simulate the virtual peer the patients can communicate with, e.g., the therapist or any other counselor (55), our approach is inspired by Craig et al. (43)) and uses a virtual human to represent the dysfunctional beliefs the patients are exposed to during intervention. Due to these conceptual differences, more research with a larger sample is required to confirm and consolidate our findings and the efficacy of our proposed intervention, and to understand the effects of virtual humans representing psychotic hallucinations (43) or, in our case, dysfunctional beliefs.

Usually, the effect size of cognitive behavioral therapies is smaller, for example, when compared with TAU than with a wait-list condition (56). For this reason, the medium to large effects of our proposed CAT-DB seem promising, as CAT-DB was sufficiently effective to reveal significant differences compared to TAU. However, it has to be taken into account that TAU was tailored to the participants to consider their specific needs (including, among others, psychopharmacological therapy and individual and group therapy as well as additional appointments if needed) and, therefore, the type of individual therapy may moderate the effects caused by CAT-DB (57). Thus, our results cannot be considered in insolation from individual effects mediated by TAU, e.g., through medication or applied group therapy. For each outcome, one could examine whether CAT-DB helps to achieve a certain level of the respective outcome that is on average not reached by patients without CAT-DB, or whether TAU simply takes longer to achieve the same size of effect. Additionally, we had eight participants (four from each group) who had already been discharged from psychiatric hospital between post-treatment and follow-up. Even if the number of discharged participants is equal for both groups, a change from a controlled environment, i.e., inpatient setting, to an uncontrolled one could increase the variance in our measures and affect the treatment responses at follow-up. Consequently, further studies at different centres, with larger sample sizes and varying therapeutic contexts, e.g., inpatients, outpatients, TAU, wait-list etc., are required to prove the effectiveness.

Our study explored short-term effects of our intervention. As pharmacological treatment using antidepressants can take weeks or even months until achieving significant effects, our intervention, for example, can bridge the gap between the start of pharmacological treatment and the actual onset of antidepressant effects. Therefore, it should reduce the high level of psychological strain to achieve early improvements of depressive symptoms (58). Our findings suggest that the first application of our intervention already positively affects the patients regarding the conviction ratings of dysfunctional and functional beliefs as well as symptom severity. These findings are promising as they imply that our proposed intervention has no significantly delayed treatment response. Nevertheless, the stability of the found effects is of great interest. Studies with further follow-up sessions could help to analyse whether depressive patients can also benefit from the intervention on a long-term perspective. In this vein, the patients' adherence still poses a major challenge for computer-based therapies that have to be addressed in future work (53).

A limitation of the present study is the operationalisation of the defined outcomes. The conviction ratings of the dysfunctional and functional beliefs are based on single items and are not instruments with verified psychometrical properties. An advantage of these items is that they are related to a person's individual dysfunctional and functional beliefs. On the other hand, it is unclear whether effects can also be found when instruments are used that measure on a more general level. Using a questionnaire with verified psychometrical properties for assessing the participants' dysfunctional cognitions such as e.g., the Cognition Checklist [(59); German version: (60)] might therefore improve further studies. Additionally, the usage of the BDI-II to operationalize depression severity is to criticize: Since the BDI-II assesses depression severity in the last 2 weeks and the participants filled in the BDI-II twice within 1 week (at pre-treatment and at post-treatment), there was an overlap between the period referred to by the BDI-II administered at pre-treatment and that referred to by the BDI-II administered at post-treatment. This problem could be solved by participants undergoing a larger number of CAT-DB sessions spread over a longer period or by using another inventory for assessing depression severity.

Regarding the formulation of the individual dysfunctional beliefs, it might have been difficult for some participants to identify their maladaptive cognitions depending on their state of mood [see (61–63)]. However, not only did the experimenter explain the basic concept of dysfunctional beliefs but the participants were also informed about the experimental procedure in written form. The consent information document included a section providing a description with five examples for dysfunctional beliefs characteristic of depressive disorders (i.e., “If someone sees how bad or stupid I am, they will leave me,” “I am inferior to all the others,” “I don't deserve to be treated decently,” “I must not be angry or upset,” and “I should be willing to give up my needs for the sake of others”). Furthermore, the potential priming as well as ongoing therapy might have also helped participants to make the associability of dysfunctional beliefs easier as indicated by the participants' feedback on CAT-DB, i.e., “dysfunctional beliefs were confusing in the beginning but became ever clearer.” To further improve the intervention procedure, it might be beneficial to visualize such beliefs, e.g., photography, drawings or classical imagery techniques, as this seems to help patients access their own dysfunctional beliefs in therapeutic settings (64, 65). It might be helpful to not just provide examples for concrete dysfunctional beliefs but also to name concrete situations in which such beliefs popup to enhance accessibility. Additionally, participants could also be instructed to keep a diary for a few days to record dysfunctional beliefs [see (66)].

It is to criticize that the shown virtual avatar was female for all participants. Individually different appearances of the virtual avatar might influence the effectiveness of the treatment. Avatar customization might be very useful to increase personal and emotional engagement and could therefore improve CAT-DB. Positive effects of avatar customization are subject of interest in research related to the gaming industry. For example, Birk et al. (67) found avatar customization led to increased effort in digital games. This is in line with Turkay and Kinzer (68), who also found that avatar customization was associated with increased identification with an avatar allowing the users to be more engaged and connected to the experience. Therefore, customized depression stimuli could be used to create more personalized and tailored computer-based therapies.

As it is commonly known that repetition enhances memory performance and positively affects the retrieval of stored information in the brain (69), our intervention is based on a repetitive exposition to three dysfunctional automatic beliefs to promote contradicting functional ones. Although an exposure to more dysfunctional beliefs is conceivable, the repetitive nature of our intervention could reduce the efficacy over time due to habituation effects. Instead of modifying dysfunctional beliefs and enhancing functional ones, the patients may simply get used to our intervention resulting in reduced levels of attention toward the stimulus and the actual task. This is in contrast to exposure therapies for the treatment of, e.g., phobias, where between-session habituation effects are considered positive (70). Our approach therefore necessitates a trade-off between an appropriate length of the intervention within as well as between sessions and the number of repetitive exposures to dysfunctional beliefs.

When addressing such a serious and sensitive illness like depression, it is crucial to investigate potential adverse effects caused by the proposed treatment. In addition, the fact that the participants were hospitalized with depression points to the severity of the illness, which in turn requires to be particularly careful when exposing participants to a repeated stressful experience. Even if the findings regarding the efficacy of our treatment seem promising on an overall level, our descriptive data showed that some patients had increased convictions ratings for dysfunctional beliefs as well as increased BDI-II scores after TAU and CAT-DB-TAU (see Table B.4 in Appendix B). Consequently, it seems that the repeated exposure to dysfunctional beliefs through our proposed treatment can also elevate depressogenic beliefs and can even cause deterioration. These additional insights confirm that the application of methods from cognitive behavioral therapy addressing maladaptive cognitions in patients with mild to severe depression necessitates on-site professional support to be able to guide the treatment and rapidly react to emerging adverse effects, such as an exacerbation of symptoms (33). As treatment responses are very individual and are determined by, e.g., a patient's severity of depression, quality of life or emotional state such as anxiety (71), negative effects are deemed as unavoidable and frequently occur even during well-delivered cognitive behavioral therapies (72). To reduce the risk of detrimental effects, we therefore recommend a sensitive application of our method with additional face-to-face meetings with a mental health professional to constantly monitor the treatment progress and early identify deterioration (73). Beside qualitative methods, quantitative instruments such as questionnaires, e.g., the Negative Effects Questionnaire (74), could be administered to quantify negative treatment responses and determine the relationship between adverse effects and treatment outcome. Regarding the outliers in our data, which were removed due to extreme ratings of the taken measures, inferential statistics without removing the outliers do not indicate any adverse effects of our intervention (see Appendix B). Nonetheless, it is important to further investigate adverse effects and deterioration rates caused by our treatment with a larger body of participants to better understand the patients' treatment responses and gain additional knowledge that contributes to a more effective and, particularly, successful application of our avatar-based intervention.

## Data Availability Statement

The raw data supporting the conclusions of this article will be made available by the authors, without undue reservation.

## Ethics Statement

The studies involving human participants were reviewed and approved by Ethics Committee of the University of Regensburg, Germany. The patients/participants provided their written informed consent to participate in this study. Written informed consent was obtained from the individual(s) for the publication of any potentially identifiable images or data included in this article.

## Author Contributions

YS was the chief investigator of the study. MK and MD implemented and designed the application and coordinated the experiment. RR, TW, CW, and CN were involved in the conceptualization and design of the study as well as the manuscript drafting. All author has substantially contributed to conducting the underlying research and drafting this manuscript.

## Conflict of Interest

The authors declare that the research was conducted in the absence of any commercial or financial relationships that could be construed as a potential conflict of interest.
