# Supplementary material for: Computer-Assisted Avatar-Based Treatment for Dysfunctional Beliefs in Depressive Inpatients: A Pilot Study
Source: Front Psychiatry. 2021 Jul 15;12:608997. doi: 10.3389/fpsyt.2021.608997 (PMC8319718; doi:10.3389/fpsyt.2021.608997)
Supplement: Supplementary file 1 [file Data_Sheet_1.pdf]

Appendix A

Table A.1

Data represent mean ± SD (standard deviation). Age, Age of Onset, Depressive episodes, Duration of index episode, Weeks since Admission, Days until Follow-Up and Scores Reached in the Pretreatment Measurements

	CAT-DB+TAU		TAU				
	M	*SD*	*M*	*SD*	*t*	*df*	*p*
Age, years	34.83	11.83	38.38	13.70	0.809	32	.425
Age of onset, years	23.27	13.46	27.08	13.50	0.745	26	.463
Depressive episodes, n	6.41	7.92	2.75	0.88	-1.291	18	.213
Duration of index episode, days	19.67	16.71	18.28	10.86	0.268	29	.791
Time since admission, weeks	4.67	3.00	3.75	2.39	-0.976	32	.336
Time until follow-up, days	15.83	3.46	15,5	2.48	-0.319	32	.752
BDI-II score	30.67	8.39	30.06	7.72	-0.218	32	.829
Conviction ratings for dysfunctional beliefs	81.74	14.77	82,73	10.22	-0.224	32	.824
Conviction ratings for alternative beliefs	45.54	30.24	57,81	17.02	1.433	32	.161

Note. BDI-II score = score reached in the German version of the Beck Depression Inventory-II. Conviction ratings for dysfunctional beliefs = mean score of the conviction ratings given for each of the participant's three dysfunctional beliefs. Conviction ratings for alternative beliefs = mean score of the conviction ratings given for each of the participant's three alternative beliefs.

Appendix B

In this Appendix we report all analyses without removing any outlier.

For the conviction ratings for the dysfunctional beliefs the 2 × 3 repeated-measures ANOVA with the between-subjects factor group (CAT-DB+TAU group, TAU group) and the within-subjects factor session (pretreatment, posttreatment, follow-up) revealed a significant effect for Session, $F(1.706, 54.597) = 22.772$, $p < .001$, $\eta^2 = .416$. No significant effects were found for the Group × Session interaction, $F(1.706, 54.597) = 1.411$, $p = .252$, $\eta^2 = .042$ and for Group, $F(1, 32) = 1.246$, $p = .273$, $\eta^2 = .037$.

For the conviction ratings of the functional beliefs the ANOVA revealed significant effects for Session, $F(1.662, 53.187) = 8.621$, $p = .001$, $\eta^2 = .21$ and for the Group × Session interaction, $F(1.662, 53.187) = 3.364$, $p = .050$, $\eta^2 = .095$. There was no significant effect for Group, $F(1, 32) = .315$, $p = .579$, $\eta^2 = .010$.

For the symptom severity the ANOVA revealed a significant effect for Session, $F(1.894, 60.612) = 16.25$, $p < .001$, $\eta^2 = .337$. No significant effects were found for the Group × Session interaction, $F(1.894, 60.612) = 1.254$, $p = .291$, $\eta^2 = .038$ and for Group, $F(1, 32) = .302$, $p = .587$, $\eta^2 = .009$.

This is a provisional file, not the final typeset article

Table B.1

Means of dependent variables at pretreatment and posttreatment by group

	Pretreatment		Posttreatment				
	M	*SD*	*M*	*SD*	*t*	*df*	*p*
Conviction ratings for dysfunctional beliefs							
CAT-DB+TAU	81.74	14.77	62.11	25.13	3.539	17	.003
TAU	82.73	10.22	71.45	21.86	2.528	15	.001
Conviction ratings for functional beliefs							
CAT-DB+TAU	45.53	30.24	57.70	30.23	-3.233	17	.005
TAU	57.81	17.01	58.87	24.42	-0.276	15	.787
Symptom Severity: Beck Depression Inventory-II							
CAT-DB+TAU	30.67	8.39	24.33	11.14	3.617	17	.002
TAU	30.06	7.71	27.5	9.76	1.275	15	.222

Table B.2

Means of dependent variables at pretreatment and follow-up by group

	Pretreatment		Follow-Up				
	M	*SD*	*M*	*SD*	*t*	*df*	*p*
Conviction ratings for dysfunctional beliefs							
CAT-DB+TAU	81.74	14.77	57.57	25.61	5.661	17	<.001
TAU	82.73	10.22	67.85	21.14	3.964	15	.001
Conviction ratings for functional beliefs							
CAT-DB+TAU	45.53	30.24	61.98	26.52	-3.828	17	.001
TAU	57.81	17.01	62.40	19.89	-1.159	15	.265
Symptom Severity: Beck Depression Inventory-II							
CAT-DB+TAU	30.67	8.39	22.28	10.24	5.343	17	<.001
TAU	30.06	7.71	24.5	8.63	2.652	15	.018

This is a provisional file, not the final typeset article

Table B.3

Means of differences of dependent variables between sessions by group

	TAU		CAT-DB+TAU				
	M	*SD*	*M*	*SD*	*t*	*df*	*p*
Conviction ratings for dysfunctional beliefs							
Pretreatment – Posttreatment	11.25	17.80	19.63	23.53	-1.159	32	.255
Pretreatment – Follow-Up	14.88	23.53	24.18	18.12	-1.619	32	.115
Conviction ratings for functional beliefs							
Pretreatment – Posttreatment	-1.06	15.42	-12.17	15.96	2.057	32	.048
Pretreatment – Follow-Up	-4.58	15.82	-16.44	18.22	2.014	32	.052
Symptom Severity: Beck Depression Inventory-II							
Pretreatment – Posttreatment	2.56	8.04	6.33	7.43	-1.421	32	.165
Pretreatment – Follow-Up	5.56	8.39	8.39	6.66	-1.094	32	.282

Table B.4

Frequencies and percentages of patients with adverse treatment responses in terms of increased conviction ratings for dysfunctional beliefs and BDI-II score between sessions by group

	TAU (n = 16)		CAT-DB+TAU (n = 18)	
	n	%	*n*	%
Conviction ratings for dysfunctional beliefs				
Pretreatment – Posttreatment	2	12.50	3	16.67
Pretreatment – Follow-Up	2	12.50	2	11.11
Symptom Severity: Beck Depression Inventory-II				
Pretreatment – Posttreatment	4	25.00	5	27.78
Pretreatment – Follow-Up	4	25.00	3	16.67

This is a provisional file, not the final typeset article
